# An in-silico assessment of efficacy of two novel intra-cardiac electrode configurations versus traditional anti-tachycardia pacing therapy for terminating sustained ventricular tachycardia

**DOI:** 10.1016/j.compbiomed.2021.104987

**Published:** 2021-12

**Authors:** Shuang Qian, Adam Connolly, Caroline Mendonca-Costa, Fernando Campos, Steven E. Williams, John Whitaker, Christopher A. Rinaldi, Martin J. Bishop

**Affiliations:** a School of Biomedical Engineering and Imaging Sciences, Rayne Institute, King's College London, 4th Floor, Lambeth Wing, St. Thomas' Hospital, Westminster Bridge Road, London, SE1 7EH, United Kingdom; b Invicro, Burlington Danes Building, Du Cane Rd, London, W12 0N, United Kingdom; c Department of Cardiology, Guy's and St Thomas' Hospital, London, SE1 7EH, United Kingdom

**Keywords:** Anti-tachycardia pacing, Ventricular arrhythmias, Computational modelling, Low-energy defibrillation, Implanted cardioverter defibrillators, Personalised electrotherapy

## Abstract

The implanted cardioverter defibrillator (ICD) is an effective direct therapy for the treatment of cardiac arrhythmias, including ventricular tachycardia (VT). Anti-tachycardia pacing (ATP) is often applied by the ICD as the first mode of therapy, but is often found to be ineffective, particularly for fast VTs. In such cases, strong, painful and damaging backup defibrillation shocks are applied by the device. Here, we propose two novel electrode configurations: “bipolar” and “transmural” which both combine the concept of targeted shock delivery with the advantage of reduced energy required for VT termination. We perform an in silico study to evaluate the efficacy of VT termination by applying one single (low-energy) monophasic shock from each novel configuration, comparing with conventional ATP therapy. Both bipolar and transmural configurations are able to achieve a higher efficacy (93% and 85%) than ATP (45%), with energy delivered similar to and two orders of magnitudes smaller than conventional ICD defibrillation shocks, respectively. Specifically, the transmural configuration (which applies the shock vector directly across the scar substrate sustaining the VT) is most efficient, requiring typically less than 1 J shock energy to achieve a high efficacy. The efficacy of both bipolar and transmural configurations are higher when applied to slow VTs (100% and 97%) compared to fast VTs (57% and 29%). Both novel electrode configurations introduced are able to improve electrotherapy efficacy while reducing the overall number of required therapies and need for strong backup shocks.

## Highlights

• Low-energy electrotherapy protocols suggest promise of reducing strong shocks.
• Our 2 novel electrode configurations specifically target the arrhythmic substrate.
• Their efficacies on terminating sustained VTs are higher than ATP.
• Our ‘transmural’ configuration requires<1 J shock energy.

## 1 Introduction

Ventricular tachycardia (VT) is an important cause of mortality and morbidity. Patients with previous VT or at risk of VT may receive an implanted cardioverter defibrillator (ICD), which automatically detects and delivers appropriate electrotherapy to terminate the VT. Although ICDs decrease the risk of sudden cardiac death (SCD) and improve mortality [1], many significant clinical challenges remain. Strong defibrillation shocks are delivered from ICDs to terminate episodes of (otherwise lethal) ventricular fibrillation (VF), as well as fast and polymorphic VTs. Such strong shocks are extremely painful and can cause long-term damage to cardiac tissue in patients receiving frequent therapies, which reduces patient quality of life (QOL) [2,3] and is associated with increased mortality [4]. Current ICDs often initially deploy anti-tachycardia pacing (ATP) in order to attempt to terminate the VT, keeping the strong defibrillation shock as backup. ATP involves the delivery of several sequences of pacing stimulus at a slightly higher rate than the VT, in order that the paced stimulus may interact with the centre of reentrant circuit to eliminate the excitable gaps that help to sustain the VT. Although ATP is found to be effective at terminating slow VTs (VT cycle length (Cl) >320 ms), it is less efficacious in fast VTs (VTCl between 240 ms and 320 ms), reported to be as low as 50–79% [[5], [6], [7]].

In addition to stimulation through an electrode directly in contact with the heart (like in ATP), cardiac tissue can also be activated by “virtual electrodes (VE)”; whereby extracellular electrodes remote from the heart cause the existence of regions of hyperpolarisation or depolarisation within the myocardium. VE formation is driven by the interaction of the strong electric field induced between the electrodes with the heterogeneity in conductivity properties between the intracellular and extracellular spaces of the myocardium [8]. The VE effect has been used to explain the efficacy of defibrillation [[9], [10], [11]].

However, the requirement for high-energy (strong) defibrillation shocks, in order to ensure efficacy, used in ICDs results in limitations, such as reducing QOL (particularly in the event of inappropriate shocks) and limited battery life [12]. These clinical concerns have recently accelerated the quest to find lower-energy electrotherapy [[13], [14], [15], [16]]. Low energy protocols aim to apply several successive electrical shocks, with relatively small strength, achieving similar efficacy to strong defibrillation shocks for ATP-refractory VT episodes [16] and fibrillation [15,17]. Despite their initial success, all protocols were delivered via conventional shock-vectors, and it remains unclear the mechanistic processes driving why the particular protocols used (often highly complex) were more efficacious than others. A significant challenge therefore remains to better understand, optimise and personalise lower-energy shock protocols for ATP-refractory VTs in infarcted hearts to facilitate translation.

For decades, computational models have been widely applied to understand the mechanism of VT occurrences [18], simulate clinically-realistic defibrillation devices [[19], [20], [21]], optimise ATP protocols [22] and also in the design of novel electrode configurations [23,24]. Modelling used in this context has significant utility in providing a carefully controlled, repeatable environment in which specific parameters may be tested and efficacies can be evaluated.

In this study, we present the use of biophysically-detailed simulation to evaluate the efficacy of two novel electrode configurations, which are suitable for patient-specific optimisation, against VT. Both configurations combine the concept of targeted shock delivery, based on *a priori* known VT substrate location, and low-energy electrotherapy. The first configuration is the “bipolar electrode” configuration, a conceptual configuration proposed by our group previously [23]. Unlike standard electrode placement in ICDs, this configuration does not have a can/ground placed outside of the heart. Instead, both positive and negative electrodes (localised in a specific alternating array) are placed inside the ventricular cavity which can provide directional stimulation to the specific region of tissue harbouring the centre of the reentrant circuit. This configuration also has the advantage of confining the electric field to the immediate vicinity of the heart, reducing the energy deposited outside of heart which is an important source of pains and discomfort in patients [25].

Secondly, we introduce a further novel configuration, termed the “transmural electrode” configuration. This, more clinically achievable and practical configuration, consists of two electrodes of opposite polarity - one intra-cavity, the other extra-cavity, producing a transmural field specifically targeted across the centre of reentrant circuit (critical isthmus (CI)). This configuration was conceived from the idea that any stimulus which simultaneously activates the CI (via the VE mechanism by applying opposite stimuli from either side) may be successful in efficiently terminating VT. This configuration aims to reduce the strength needed for electrotherapy by applying CI-targeted shocks.

These electrode configurations (bipolar and transmural) will be embedded within a cohort of high-resolution left ventricular (LV) porcine models constructed from *in vivo* magnetic resonance imaging (MRI) with a 1 mm isotropic resolution (not available clinically) to assess their efficacy against stable monomorphic VT. Specifically, models will be used to test the hypothesis that such targeted electrotherapy, delivered towards (bipolar) or ‘through’ (transmural) the scar substrate will terminate sustained VTs with greater efficacy compared to standard ATP and requiring considerably less than standard defibrillation shocks.

## 2 Methods

### 2.1 Geometrical models and electrode configurations

**Fig. 1:**
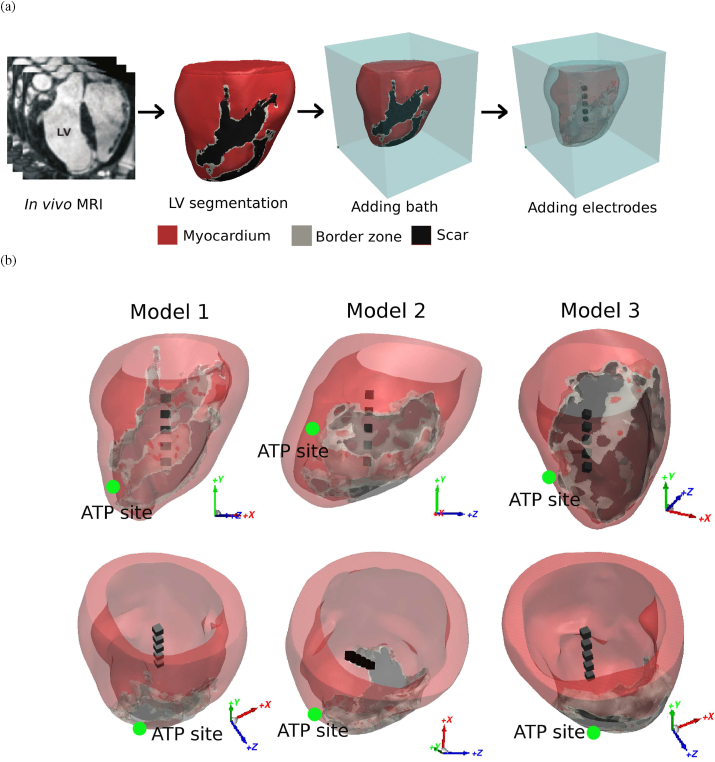
(a) Model generation pipeline of porcine LV meshes from *in vivo* MRI. (b) Placements of novel bipolar electrodes in the LV models, viewed from side and top. ATP delivery sites in all three LV models are marked with green dots which replicate the delivery site in clinical ICDs (apical septum of right ventricle). (For interpretation of the references to colour in this figure legend, the reader is referred to the Web version of this article.)

A cohort of infarct porcine finite element (FE) LV models were developed based on late-gadolinium enhanced cardiovascular MRI obtained seven weeks following myocardial infarction, as shown in Fig. 1(a). Full details of the pipeline for image-based model construction has been described previously [18]. Briefly, images were segmented semi-automatically in the open source package Seg3D (www.seg3d.org) to delineate blood pool, myocardium, infarcted scar and border-zone (BZ) [26]. Tetrahedral FE meshes were created directly from the segmented images using CGAL library (https://www.cgal.org/), and smoothed using Meshtool [27]. All LV models were immersed within a cubic extracellular bath, as shown in Fig. 1(a). Fibre architecture was incorporated using a rule-based approach [28].

The spacing between the electrodes are chosen to be the same as the length of the cubic electrodes which is because our previous study shows the total electrical flux is insensitive to the spacings of the bipolar electrodes [23] The arrangements and placements of the bipolar electrode configuration in all three LV models are shown in Fig. 1(b). The electrode geometries were defined with Simpleware (Version P-2019.09; Synopsys, Inc., Mountain View, USA), constituting five cubes of size 4 × 4 × 4 mm^3^ with 4 mm spacing, which is based on [23], but enlarged by a factor of 4 due to the smaller rabbit ventricles were used in this previous study. The spacing between the electrodes were chosen to be the same as the length of the cubic electrodes, as our previous study showed that the total electrical flux is insensitive to the spacings of the bipolar electrodes [23]. The bipolar electrodes were placed approximately in the middle of the LV cavity, aligned vertically, in order to enable its ability of target any area in the LV by switching the polarities of the surfaces [23]. In addition, they are placed sufficiently far from endocardial boundaries to minimize boundary effects. One of faces in the electrodes are orientated towards the scar region to provide targeted shock delivery. Following electrode placements, models were re-meshed in Simpleware to produce final average edge lengths of the 330 μm ± 25 μm for myocardium, 600 μm ± 10 μm for bath and 370 μm ± 10 μm for bipolar electrodes. Note, the bipolar electrodes were defined as cubic regions within the bath space, and did not require specific re-meshing.

### 2.2 Biophysical model

Electrophysiological dynamics of the LV models were simulated using the Cardiac Arrhythmia Research Package [29] (https://carpentry.medunigraz.at/). The ventricular myocardium was represented by the bidomain equations [30]:

(1) $$\nabla \cdot \left(\sigma_{i}\nabla \varphi_{i}\right)=\beta I_{m},$$

(2) $$-\nabla \cdot \left(\sigma_{e}\nabla \varphi_{e}\right)=\beta \left(I_{m}-I_{e}\right),$$

where *σ*_*i*_ and *σ*_*e*_ are the intra- and extracellular conductivities, *φ*_*i*_ and *φ*_*e*_ are the intra- and extracellular electrical potentials, *β* is the surface area of membrane contained within a unit volume equal to 0.14 μm^−1^, *I*_*m*_ is the transmembrane current density and *I*_*e*_ is the extracellular stimulus current density. The transmembrane current density is given as:

(3) $$I_{m}=C_{m}\frac{\partial V_{m}}{\partial t}+I_{ion}\left(V_{m},\eta \right),$$

where *C*_*m*_ is the membrane capacitance per unit area, *t* is time and *I*_*ion*_ is the ionic current density depending on *V*_*m*_ and *η* which is a vector of state variables describing channel gating and ionic concentrations. The transmembrane potential *V*_*m*_ is:

(4) $$V_{m}=\varphi_{i}-\varphi_{e}$$

The extracellular bath is described as:

(5) $$\nabla \cdot \left(\sigma_{b}\nabla \varphi_{b}\right)=0,$$

where *σ*_*b*_ is the extracellular bath conductivity and *φ*_*b*_ is the electrical potential in the bath space.

No flux conditions are imposed at the tissue and extracellular bath boundaries. while the flux is continuous between *φ*_*e*_ and *φ*_*b*_:

(6) $$n\cdot \left(\sigma_{b}\nabla \varphi_{b}\right)=n\cdot \left(\sigma_{e}\nabla \varphi_{e}\right),$$

(7) $$\varphi_{b}=\varphi_{e}.$$

At the boundaries of extracellular bath, no flux condition is imposed.

Full bidomain simulations were performed during the electrotherapy delivery. During pre-conditioning (post-shock simulation), the more computationally efficient monodomain model was used:

(8) $$\nabla \cdot \left(\sigma_{m}\nabla V_{m}\right)=\beta \left(I_{m}-I_{s}\right),$$

where *σ*_*m*_ is the harmonic mean conductivity tensor.

Anisotropic conductivity was assigned to reproduce the characteristic properties of myofibres. For the healthy tissue, the intra- and extra-cellular conductivities along the fibre direction were *σ*_*il*_ = 0.174 and *σ*_*el*_ = 0.625 *S*/*m*, while the intra and extra-cellular conductivities transverse to the fiber are *σ*_*it*_ = 0.019 and *σ*_*et*_ = 0.236 *S*/*m* [31]. Based on our previous VT induction work [18], the conductivity for BZ tissue was set to be half of the conductivity for healthy tissue to simulate conduction slowing due to gap junction uncoupling and fibre disarray. The scar conductivity was set to 0.05 *S*/*m*, while the bath conductivity was set to 1 *S*/*m*, based on previous works [32,33].

Ionic membrane dynamics (*I*_*ion*_) in the porcine models was represented by Ten Tusscher (TT) model [34]. Based on ours previous works [18], the ionic properties in the health tissue were adjusted including 50% increase of the potassium currents *I*_*Kr*_ and *I*_*Ks*_ from the default settings of TT model to more accurate replicate the shorter porcine ventricular action potential duration (APD). In order to produce the substrates for block and reentry in the infarcted ventricle, the ionic properties in BZ were altered from health tissue including a 30%–65% reduction of the conductances of the potassium currents *I*_*Kr*_ and *I*_*Ks*_, and 10%–90% reduction of the conductances of the sodium current *I*_*Na*_ based on ours [18] and other previous computational studies [22].

### 2.3 VT induction

Prior to tissue simulations, the single cell TT model was paced for 100 cycles at 2 Hz in order to produce stable single-cell states. These were saved and used as initial conditions for tissue simulations. Each LV model was paced either at the LV base or the LV apex in order to induce VT [18]. This involved three stimulus S1 at a basic cycle length of 500 ms, followed by S2 with a shorter coupling interval, reducing sequentially by 10 ms from 500 ms until unidirectional block was observed. If failure to capture occurred prior to unidirectional block occurring, another premature stimulus S3 was added which follows the same process until a block was achieved.

Following unidirectional block, in order to maintain the induced VT, the ionic properties in the BZ were adjusted to be the same as the ionic properties in the healthy tissue (as we have shown that longer APD in the BZ is in fact anti-arrhythmic, attenuating sustenance [18]). In addition, the conductivities in healthy tissue and BZ were adjusted, without altering their conduction velocities by more than 60% [22]. All induced VTs were then run for 3 cycles to ensure they were monomorphic and stable. After that, in order to generate a number of VT episodes with various VT cycle lengths (VTCLs), we adjusted conductivities and ionic properties in BZ as in Refs. [18,22]. The ionic properties in the BZ were altered including a 30%–65% reduction of the conductances of the potassium currents *I*_*Kr*_ and *I*_*Ks*_, and 10%–90% reduction of the conductances of the sodium current *I*_*Na*_. The conductivities were adjusted without varying conduction velocity by more than 10%. Each VT episode was simulated for more than five cycles to ensure it was stable and monomorphic before applying electrotherapy.

**Fig. 2:**
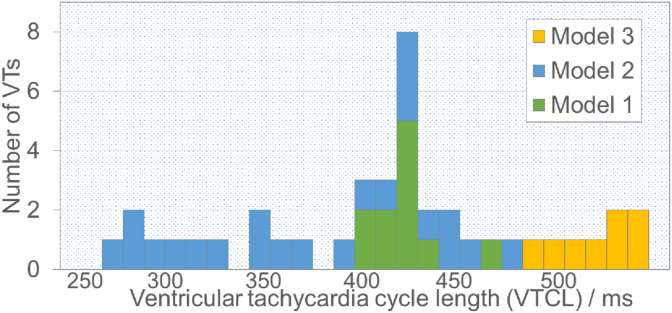
The ventricular tachycardia cycle lengths (VTCLs) histogram.

In total, 40 separate VT episodes were generated within three LV models with their VTCLs ranging from 260 ms to 530 ms as shown in Fig. 2, including 7 fast VTs (VTCL ≤320 ms) and 33 slow VTs (VTCL > 320 ms). Next, three different electrotherapy protocols were applied to each of the 40 VT episodes independently and their efficacy evaluated. Specifically, ATP was compared to single shocks delivered from the bipolar electrode configuration and from the transmural electrode configuration.

### 2.4 ATP protocols

Traditional burst ATP therapy was delivered from a septal location near the apex of the LV (RV side), as shown in Fig. 1(b). Note that the scar regions in all three LV models are located proximal to the ATP delivery site. The burst ATP protocol consisted of 2 sequences of 8 pacing pulses with an equal inter-stimulus interval pacing at 88% of the VTCL. In the 2nd sequence, the pacing interval was decreased by 10 ms. The initial pacing start timing was calculated as the time at 88% of the VTCL after the last activation was detected at the pacing location. The stimulus current was 50 μA/cm^2^, delivered to a volume of 5 × 5 × 5 mm^3^, replicating the pacing delivered from the ICD lead.

### 2.5 Bipolar/transmural electrode configurations and electrotherapy protocols

**Fig. 3:**
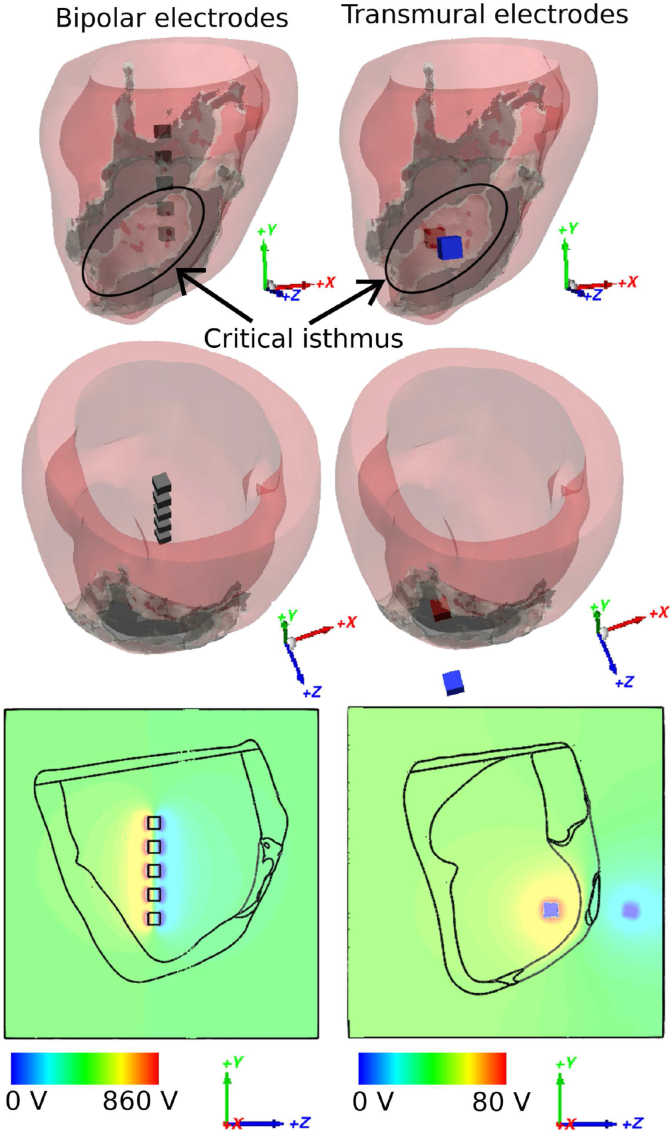
Bipolar and transmural electrode configurations and field distributions. Bipolar electrode (left, black) and transmural electrode (right, blue and red) configurations in Model 1 shown in side and top views. The clipping images show the extracellular potential distribution during stimulus delivery. (For interpretation of the references to colour in this figure legend, the reader is referred to the Web version of this article.)

Both the bipolar and transmural electrode configurations simulated here are designed to deliver the stimulus to the centre of the re-entrant circuit, through the CI to perform targeted electrotherapy, as shown in Fig. 3. In the bipolar electrode configuration, the ground electrode (0 *V*) was assigned to the faces near the CI, and applied voltages assigned to the opposite faces, as shown in the clipping images of Fig. 3. As discussed previously, the bipolar configuration constitutes five cubes of size 4 × 4 × 4 mm^3^ with 4 mm spacing [23], aligned vertically, approximately in the middle of the LV cavity. In the transmural configuration, the positive electrode is assigned to be inside the LV blood pool, and ground outside of LV within the RV blood pool. The transmural electrodes consist of two cubic electrodes with the side-length 5 mm. Both electrodes are placed close to the centre of the CI with the minimum distance to cardiac tissue of approximately 1 cm.

The electrotherapy protocols of both novel electrode configurations were first tested on a smaller cohort of 6 VTs, in order to choose an optimal stimulus delivery time and minimum strength needed for terminating VT. The duration of the stimulus applied via both transmural and bipolar configurations was fixed at 5 ms and the strength varied in steps of 20 *V* in the bipolar case and steps of 10 *V* in the transmural case. The stimulus was applied at different timings, defined as a percentage of the VTCL since the last activation detected at the CI. Based on the success of electrotherapy applied by each respective configuration upon the 6 initial VT episodes, we chose an optimal electrotherapy setup in terms of delivery time and strength. Specifically, two strengths were chosen in order to compare the two novel configurations with different goal efficacies: the first strength (termed *S*_pEff_) was defined as the stimulus strength which was seen to achieve a partially high efficacy at 5 out of 6 of the tested VTs; the second strength (termed *S*_fEff_) was defined as the stimulus strength which was seen to achieve full efficacy of 6 out of 6 of the tested VTs.

### 2.6 Sensitivity of transmural electrode configuration

**Fig. 4:**
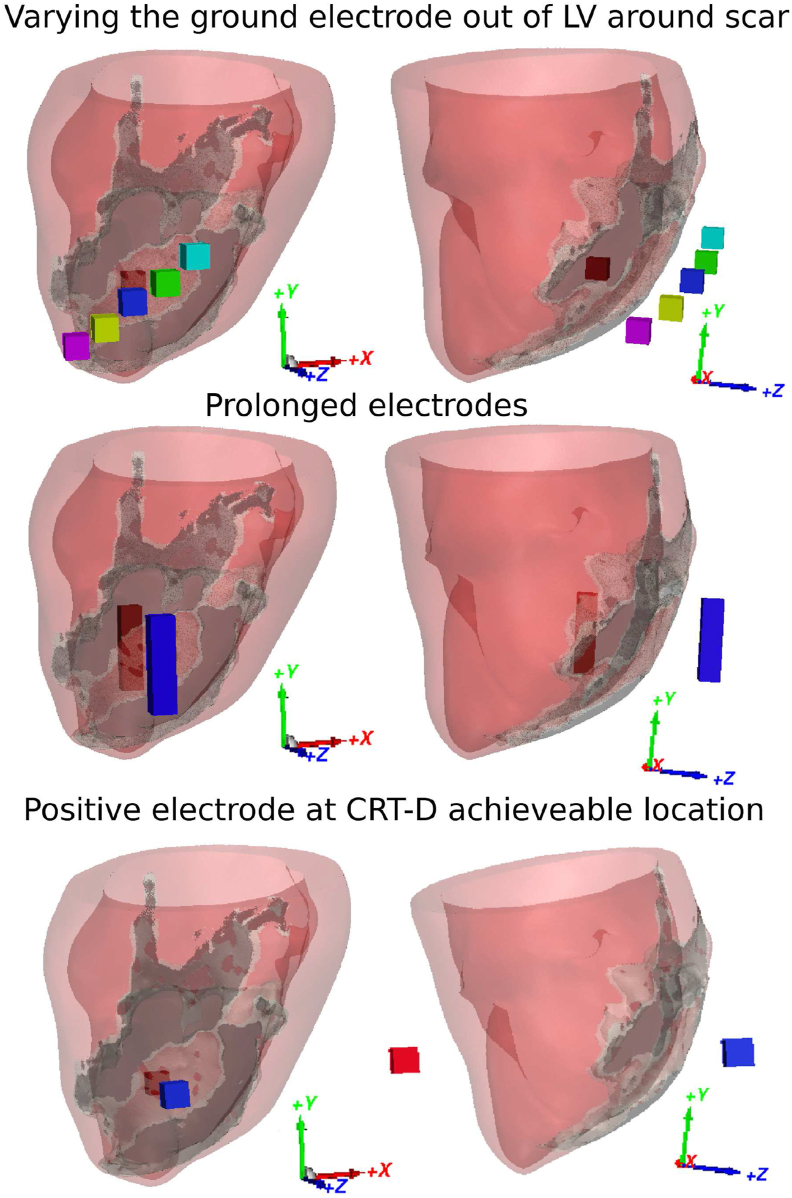
Sensitivity tests setup of the transmural electrode configurations shown in direct (left) and side (right) views. First test (top) varies the location of the ground electrode (outside the LV) in 5 different locations. The middle blue electrode represents the same location as the default setup. Second test (centre) shows the prolongation of the transmural electrodes. Third test (bottom) shows the movement of the positive electrode to be outside of the LV cavity, which maintains a shock vector through the CI. (For interpretation of the references to colour in this figure legend, the reader is referred to the Web version of this article.)

The practical clinical translation of the transmural electrode configuration proposed here may encounter alterations, such as viability in the specific placement of electrodes, electrode size. We therefore conducted three sensitivity tests of shock vectors in the transmural electrode configuration, as shown in Fig. 4, applied to a smaller cohort of three VTs (one from each LV model). The first test was designed to vary the locations of electrodes with respect to the scar locations whereby the positive electrode inside the cavity remains fixed, whilst the ground electrode outside of LV was varied to five locations in three models, covering all regions along the length of the CI, giving 15 scenarios. Note that the middle location, shown in blue in Fig. 4, is same as the setup used in the default setup.

The second test was designed to increase the size of both electrodes, more similar to the length of the RV coil in clinical ICDs. As is shown in Fig. 4, both electrodes were prolonged by 20 mm, and their placements were adjusted accordingly to make sure the centroids of both electrodes were still in line with the centre of the CI, giving 3 scenarios.

The final test involved moving the positive electrode (originally placed inside the LV blood pool) to now be outside of the LV, while maintaining the shock vector to be still through the CI. This new location may be more representative of a potential shock vector between an RV electrode and LV coronary sinus electrode in a clinical cardiac resynchronization therapy defibrillator (CRT-D) device.

In all tests, a single stimulus using *S*_fEff_ was applied to all three VTs tested.

## 3 Results

### 3.1 Dynamics of induced VTs

**Fig. 5:**
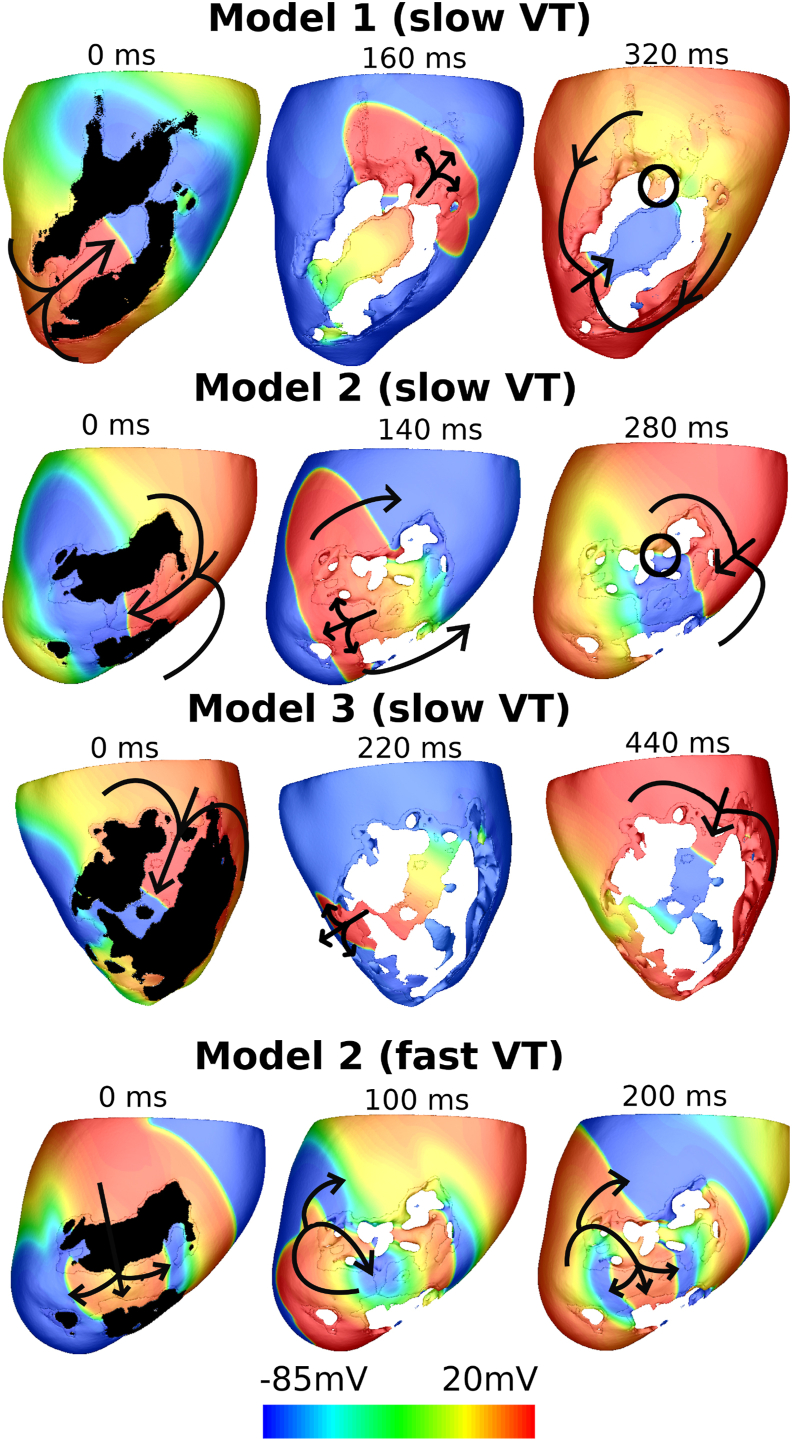
Transmembrane potential (*V*_*m*_) maps of at different timings during slow VTs induced in Model 1, 2 and 3, along with a fast VT induced in Model 2. VTCLs are 400 ms, 350 ms, 530 ms and 270 ms. Only the first time plots show the scar region while the others are removed in order to clearly show the pathway of activation wave. The black arrows show the propagation direction of the VT through the CI. The black circles mark the small extra pathways at the exit of the CI in Model 1 and 2.

Three example of slow VTs in each model and one fast VT in Model 2 are shown in Fig. 5. The VTCL of Model 3 is seen to be long because of the slow conduction in the CI which, in this model, represents a relatively long, thin anatomical pathway, combined with the fact that the entire CI region in Model 3 is BZ, unlike the other two models in which the CI regions are largely composed of healthy myocardium. The fast VT in Model 2 has a much shorter VTCL which is due to the correspondingly shorter VT reentrant pathway. As shown in Fig. 5, comparing with the slow VT in Model 2, the fast VT circulates in approximately half of the CI.

### 3.2 Optimal electrotherapy protocols for bipolar and transmural electrode configurations

**Fig. 6:**
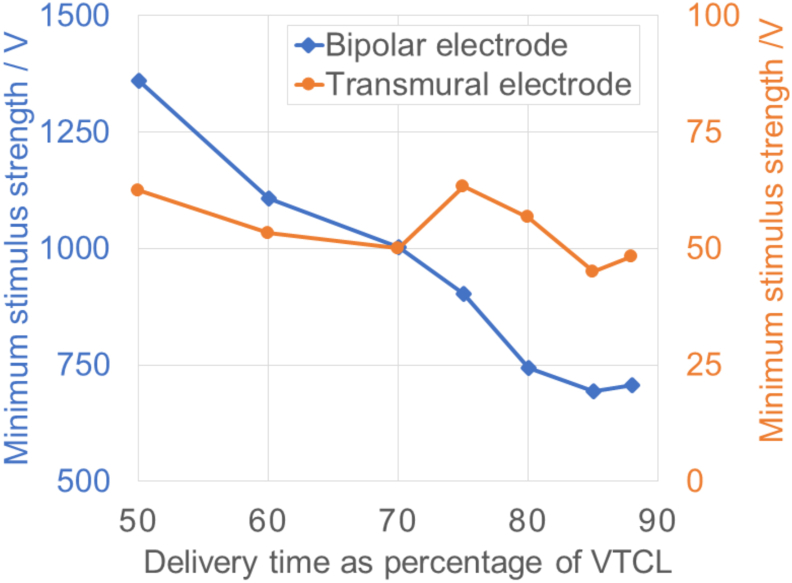
Average minimum stimulus strength needed, for terminating VT with the bipolar (left blue line) and transmural (right red line) electrode configurations, is plotted against the shock delivery time defined as the percentage of the VTCL since the last activation detected at the CI. Note that at 50% and 60% of VTCL, some VTs failed to terminate with the maximum delivered stimulus. In these cases, the average value is calculated by eliminating those unsuccessful cases. (For interpretation of the references to colour in this figure legend, the reader is referred to the Web version of this article.)

Different delivery timings and shock strengths were applied to a smaller cohort of 6 VT cases using both the transmural and bipolar electrode configurations in order to find the optimal delivery protocol for each case, as described in the Methods 2.5. As shown in Fig. 6, for the bipolar configuration (blue line), with increasing delivery time (later from the timing of the last activation), the average minimum shock strength needed for terminating (all 6) VTs decreases rapidly. At the latest delivery time of 88% VTCL (since the last sensed activation), the smallest average strength is achieved at 700 *V*. However, the effect of pacing times on the minimum shock strength in the transmural configuration (red line) is more subtle. The average strength at different delivery timings ranges from 45 *V* to 63 *V*, with a small variation of less than 20 *V*. The full results detailing the individual strengths required to terminate all 6 VTs are shown in Appendix Fig. S1.

Based on Fig. 6, we decided to use a delivery timing of 88% of the VTCL in both electrode configurations moving forward, as the average shock strength needed at this delivery time is relatively lower than the other times, and is also the same as used in ATP therapy. As shown in Appendix Fig. S1(a), application strengths *S*_pEff_ and *S*_fEff_ are 860 and 1700 *V* for the bipolar configuration, whereas for the transmural configuration are 80 and 120 *V* as in Appendix Fig. S1(b).

**Fig. 7:**
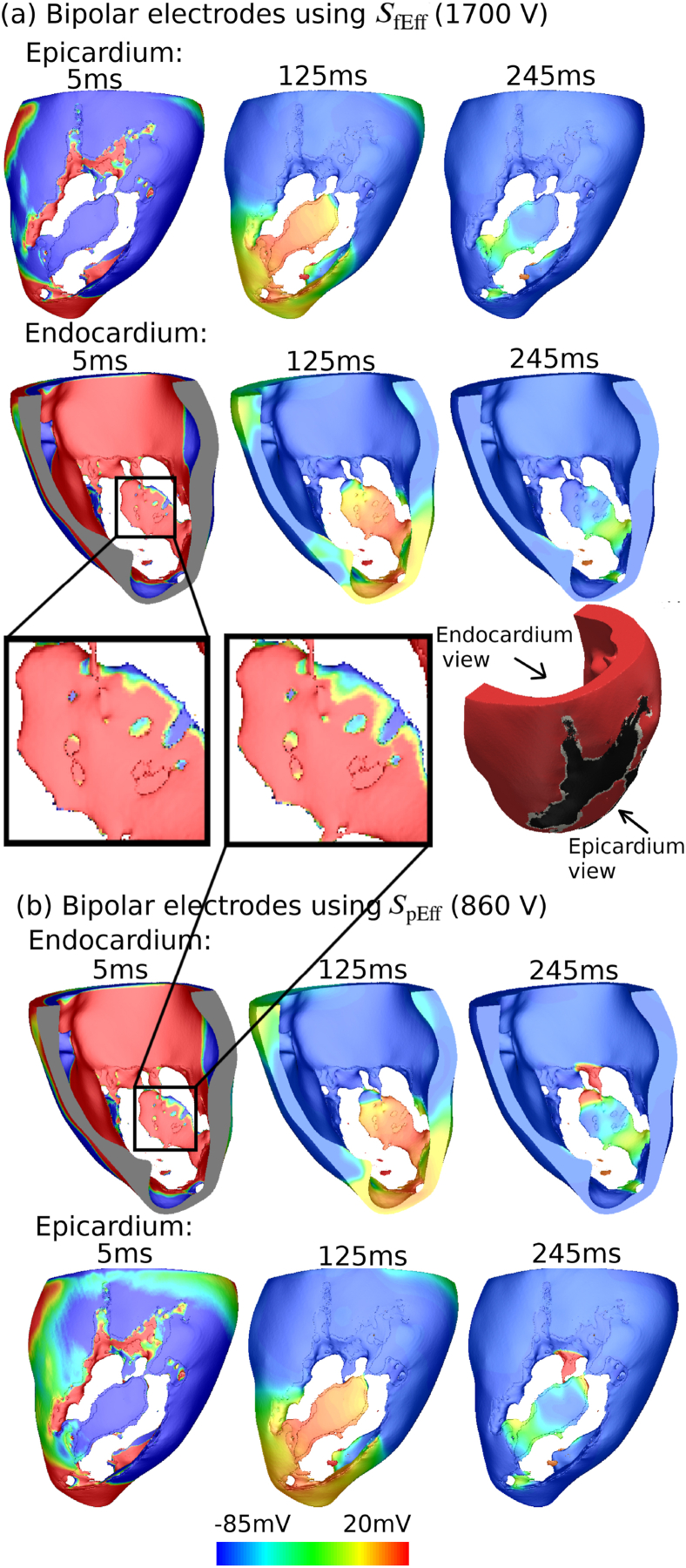
Transmembrane potential (*V*_*m*_) distributions during shock application with the bipolar electrode configuration showing successful (panel a) and unsuccessful (panel b) VT termination using strength *S*_fEff_ and strength *S*_pEff_, respectively in Model 1. Note that the stimulus applied by the bipolar electrodes starts at 88% VTCL in the CI location, as shown in Fig. 3 and lasts for 5 ms. Two views are shown from the epicardium and the endocardium of the LV. Enlarged views of the CI region are marked by blacked rectangles.

Fig. 7(a) highlights the mechanism by which a VT is terminated by the bipolar configuration using the larger shock strength *S*_fEff_ (1700 V), compared with the same VT that is not terminated by using the weaker strength *S*_pEff_ (860 V). In both cases, the Figure shows that at time 5 ms when the stimulus just ends, most of the area at the endocardium around the CI is depolarized, while most of the area at the epicardium near the CI is hyperpolarized. However, as shown in the two enlarged images, there is less depolarized tissue area seen on the endocardium within the CI in Fig. 7(b), compared to Fig. 7(a) because of the relatively smaller stimulus strength in this case. Consequently, the tissue near the tail of the CI remains excitable, so at time 245 ms it becomes depolarized by a remnant VT wavefront and the VT is not terminated.

**Fig. 8:**
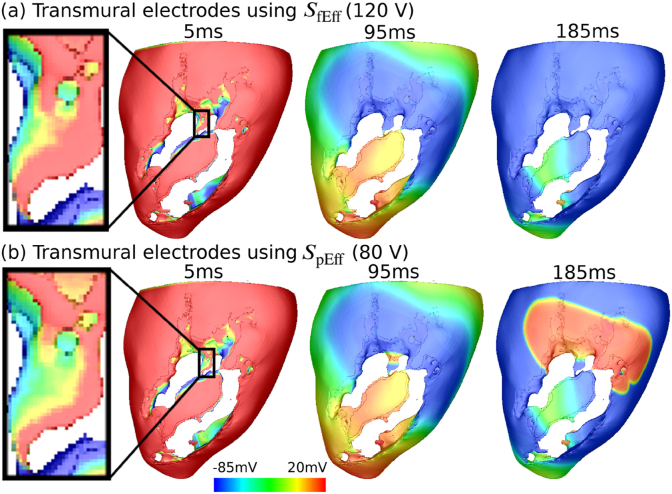
Transmembrane potential (*V*_*m*_) distributions during shock application with the transmural electrode configuration showing successful (panel a) and unsuccessful (panel b) VT termination using strength *S*_fEff_ and strength *S*_pEff_, respectively in Model 1. Note that the stimulus applied by the tranmural electrodes starts at 88% VTCL in the CI location, as shown in Fig. 3 and lasts for 5 ms. Two views are shown from the epicardium and the endocardium of the LV. Enlarged views of the CI region are marked by blacked rectangles.

Fig. 8(a) highlights the mechanism by which a VT is terminated by the transmural configuration using the larger shock strength *S*_fEff_ (120 V), compared with the same VT that is not terminated by using the weaker strength *S*_pEff_ (80 V). In both cases, at time 5 ms a large area of epicardium near the top of the CI is depolarized due to the stimulus. As shown in the two enlarged images, the larger stimulus strength in Fig. 8(a) results in depolarisation of more area at the small pathway at the mouth of the CI which successfully prevents the propagation of excitation from this pathway, terminating the VT. In contrast, the weaker strength of stimulus in Fig. 8(b) leaves a larger excitable area within this critical area, which is not sufficient to block the remaining VT wavefronts, and reentry continues.

### 3.3 Efficacy of two novel electrodes configurations comparing with ATP

**Fig. 9:**
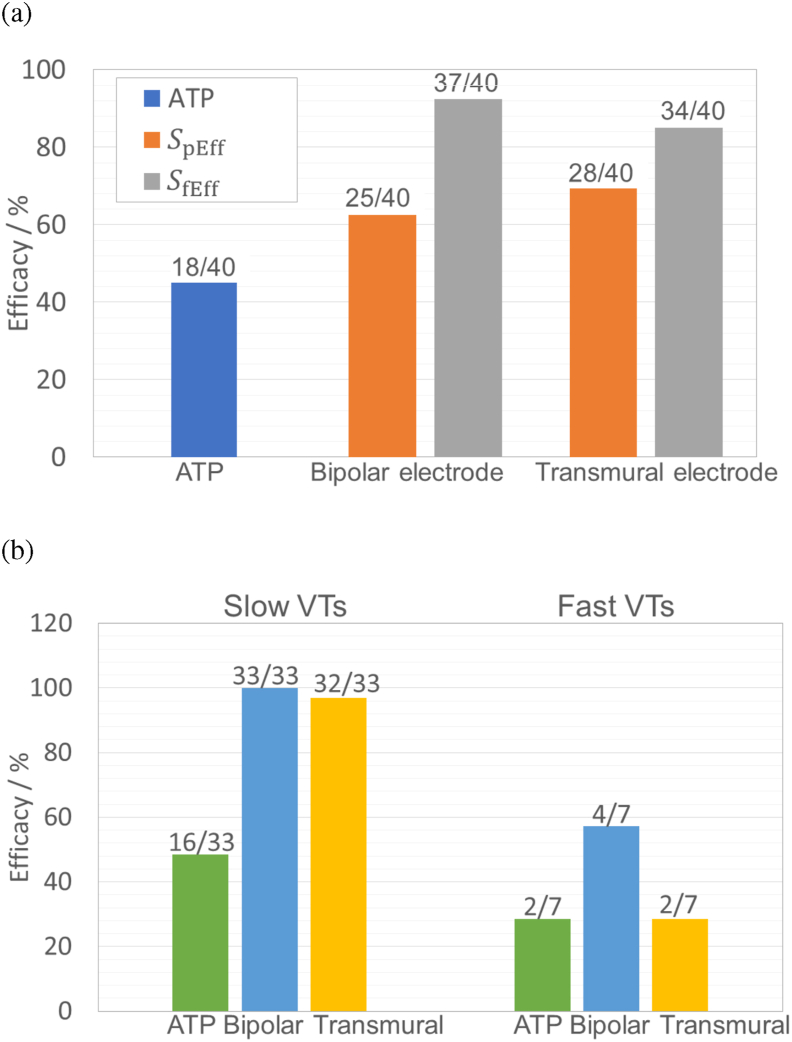
(a) Comparison of the efficacy of the bipolar and transmural electrode configurations with ATP, applied to all 40 VT episodes. Strengths *S*_pEff_ and *S*_fEff_ for bipolar configuration are 860 V and 1700 V. Strengths *S*_pEff_ and *S*_fEff_ for transmural configuration are 80 V and 120 V. (b) Comparison of the efficacies of bipolar and transmural electrodes using strength *S*_fEff_ with ATP, on slow and fast VTs separately.

Fig. 9(a) shows the efficacy of the bipolar and transmural electrode configurations for both shock strengths (*S*_fEff_ and *S*_pEff_) applied to all 40 VT episodes, in comparison with success of ATP. The Figure shows that the efficacy of both electrode configurations using either stimulus strength is higher in comparison to the efficacy of ATP (45%). Furthermore, the efficacy of both novel configurations is seen to improve with the increase of shock strength, *S*_fEff_ vs *S*_pEff_. The efficacy of the bipolar configuration is increased from 63% using strength *S*_pEff_ (860 V) to 93% using strength *S*_fEff_ (1700 V), while the efficacy of the transmural configuration is increased from 70% using strength *S*_pEff_ (80 V) to 85% using strength *S*_fEff_ (120 V). Efficacies of bipolar and transmural configurations using strength *S*_fEff_ on slow and fast VTs are compared in Fig. 9(b). The Figure shows that both novel electrode configurations are more efficient at terminating slow VTs, compared to fast VTs. The efficacy for slow VTs is seen to be as high as 100% for the bipolar configuration and 97% for the transmural configuration. Furthermore, the efficacy of the bipolar configuration for fast VTs (57%) is seen to be higher than for the transmural configuration (29%).

### 3.4 Sensitivity analysis for transmural electrode placements and geometries

**Fig. 10:**
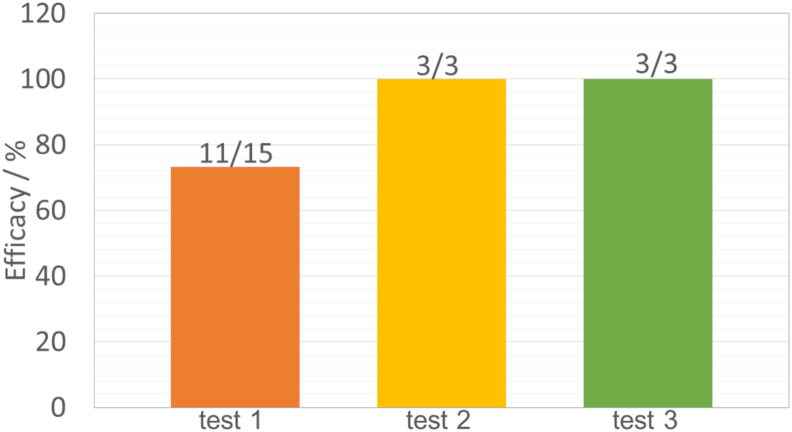
Sensitivity analysis of the specific placements and geometry of the transmural electrode configuration, tested on a smaller cohort of 3 VT episodes (one from each LV model). Test 1 varies the placement of the ground electrode outside of the LV to five locations around the CI (5 cases). Test 2 prolongs the electrodes size (3 cases). Test 3 moves the positive electrode outside of the LV cavity, whilst maintaining the shock vector through the CI (3 cases). All stimulus strengths used here are *S*_fEff_.

Three sensitivity tests examining the specific placements and geometries of the transmural configuration (as described in the Methods 2.6) were conducted on a smaller cohort of three VTs (one from each LV model). The respective efficacies for terminating VTs for each variation are shown in Fig. 10. The Figure shows that varying the placement (test 1) of the ground electrodes at the outside of the LV results in a slight reduction of efficacy to 73%, comparing with the efficacy of previous fixed placements of the transmural electrodes (85%). The Figure also shows that the efficacy of the transmural configuration is relatively insensitive to a change in electrode geometry (test 2) or moving the positive electrode outside of the LV cavity (test 3), as the efficacies of these tests remain high at 100%.

## 4 Discussion

In this virtual study, we tested the efficacy for VT termination of two novel electrode configurations (bipolar and transmural) and compared with conventional ATP using a cohort of infarcted LV porcine models. In our previous work [23], we introduced the novel bipolar electrode configuration and explored its effect on stimulating passive myocardial tissue in realistic (rabbit) cardiac models. In this study, we further assess its electrotherapy efficacy, in addition to our new transmural electrode configuration, using a comprehensive library of sustained VT episodes and compare against traditional ATP. Here, we have demonstrated that both configurations are highly effective at terminating VTs. Specifically, we demonstrate that the efficacy of bipolar and transmural configurations are 93% and 85%, higher than 45% for ATP. Both novel electrode configurations are able to provide infarct-targeted electrotherapy, focussing the applied field on the core of the reentrant circuit sustaining the VT, consequently minimising the energy required for VT termination and potentially reducing pain due to extra-cardiac stimulation. Importantly, we highlight the mechanism of VT termination by both novel electrode configurations (Fig. 7, Fig. 8) to be targeting the core of re-entrant circuit (the CI) causing depolarisation which results in functional block that stops the propagation of the VT. Our findings reveal the significant potential of these low-energy targeted electrotherapy strategies, guiding future pre-clinical or clinical testing and development.

### 4.1 Utility of our virtual ‘library’ of simulated VT episodes

The VTs in this study were induced by similar protocols used in other recent computational modelling studies [18,22]. Similar to these other works [22], in our simulations we found that the fast VTs were difficult to induce, as the excitable gap is small in such cases and the VTs are often induced but self-terminated. Interestingly, we found that the VTCLs varied significantly between models, which was mainly seen to be dependent on the explicit infarct anatomies and the CI. As shown in Fig. 2, the VTCLs in Model 3 are generally longer than the other two models, which appears to be largely driven by the long CI of Model 3 which is mainly composed of slow conducting BZ tissue (Fig. 1, Fig. 5). It is also shown in Fig. 5 that multiple pathways through the scar in Model 2 may generate ‘short-circuit’ effects leading to both fast and slow VTs in the same model. This demonstrates how the complex structure of the scar may dictate the specific VT substrate, and emphasises the utility of our high-resolution pre-clinical model cohort in being able to simulate these important aspects. Considering the VTCLs of our induced VTs are within the range of clinically recorded VTs, the variation of VTCLs between models, and the detailed representation of the VT substrate, provides a robust library of VT episodes with which to test electrotherapy efficacy.

### 4.2 Mechanistic dependence of novel electrotherapy configurations upon stimulus timing

Current ATP protocols use a variety of pacing times, ranging from 75% to 91% of VTCL [5,35,36], with little mechanistic understanding regarding the optimal value. Therefore, we conducted a grid search of the minimum stimulus strength required for VT termination for different pacing times (% of VTCL) for our bipolar and transmural electrode configurations.

We found a descending trend of minimum strength as the application times increase for the bipolar configuration (Fig. 6). A much larger shock is required if the application time is too early, as more targeted tissue is not fully repolarised. However, applying the stimulus at timings later than 80% of VTCL showed little difference in the strength required. In contrast, for the transmural configuration, little correlation between stimulus time and the minimum strength required for VT termination was found (Fig. 6).

In comparison to the bipolar electrode configuration, the transmural electrodes are situated much closer to the centre of CI and both electrodes are placed at opposite sides of the CI, which enables delivery of more energy to the targeted tissue (i.e. through the volume of the CI). We postulate that the transmural current path setup by the transmural electrode configuration is crucial to its success, inducing as it does, a strong virtual electrode response from the epicardial and endocardial tissue of the CI. Furthermore, the more concentrated field induced by the transmural configuration results in a stronger localised tissue response within the CI, enough to override the difference in the area of repolarised tissue due to pacing times. Finally, it is also interesting to note that the range of strengths needed for different VT episodes are large: up to 1500 V in the bipolar and 120 V in the transmural configuration. This suggests that personalising electrotherapy based on the prior information of anatomical structure and electrophysiology properties could improve efficacy of electrotherapy and QOL by deceasing unnecessary high shock voltage.

### 4.3 Energy requirement for novel configurations

The Defibrillation Threshold (DFT) is a conventional clinical metric in assessing ICD efficacy, which can be defined as the lowest energy required to successfully defibrillate. Current ICDs are usually programmed to deliver a first shock of 20–35 *J* energy, with additional stronger shocks if the first attempt fails [37,38]. The maximum defibrillation energy varies in patients and devices, typically being somewhat higher in subcutaneous ICD devices, but can reach up to 360 *J* [39]. To allow direct comparison with current ICDs, we estimated the approximate energy required for successful therapy for our two novel electrode configurations using similar methods as in our previous study [21] (based-on capacitive discharge): the energy required for the bipolar configuration is approximately 37 *J* for 860 *V* shock yielding 63% efficacy and 145 *J* for 1700 *V* shock yielding 93% efficacy. Although this energy is slightly higher than the current conventional ICDs, it should be noted that unlike the conventional ICDs with an extra-cardiac can placed under the clavicle, this configuration has all electrodes placed within the LV cavity. The electric field generated by these electrodes also decays rapidly with the increasing distance from the source. Consequently, the majority of the regions of high electric field are confined within the LV, thus, the energy delivered by the device (which is higher than standard ICD programming), is confined within the heart (where it is needed), potentially reducing the pain in patients due to stimulation of extra-cardiac tissues and organs.

However, the energy required for the transmural electrode configurations is 0.32 *J* for 80 *V* shock yielding 70% efficacy which increases to 0.72 *J* for 120 *V* shock yielding 85% efficacy, which is two orders of magnitude *lower* than conventional ICDs. In the transmural electrode configuration, the positive and ground electrodes are placed across the centre of reentrant circuit (CI) and also very close to each other which results in a short current path. The resulting low impedance and high voltage gradient generates more stronger VE effects, and importantly, focussed to the critical region of tissue to facilitate interruption of the reentrant circuit. Thus, only a small amount of energy is required for VT termination.

Moreover, we also show that both novel electrode configurations are more efficient, as only one single shock/stimulus is required to achieve a higher efficacy of VT termination comparing with the traditional ATP, in which multiple pacing stimulus and even multiple sequences of stimulus are required.

### 4.4 Efficacy for fast VTs

ATP is known to be found less efficient for fast VTs, compared with slow VTs, which we also found in this study. Interestingly, we also found a similar result of both novel electrode configurations. Both novel configurations were seen to be more efficient on slow VTs (efficacy of 100% and 97% for bipolar and transmural configurations, respectively), while the efficacy on fast VTs was much lower (57% and 29% in bipolar and transmural configurations, respectively). Interestingly, the bipolar configuration significantly outperformed the transmural configuration for fast VTs, whereas the transmural configuration performed similar to ATP in this case.

Fast VTs are known to have a smaller excitable gap, compared to slower VTs, making it harder for ATP to both capture and also successfully penetrate and close this gap for VT termination. The same in true for the novel electrode configurations, particularly for single shocks, as delivered here, which explains the significantly reduced efficacy in our novel configurations when applied to fast VTs (Fig. 9). In this work, we presented use of one single monophasic shock delivered from both novel electrodes configurations, as this was effective for initial evaluation of efficacy and more effective dissection of the mechanisms of terminating VTs. This is in contrast to recent low-energy electrotherapy research involving multiple shocks/stimulus in rather complex and non-intuitive protocols [14,16]. Moving forwards, our configurations may be used in conjunction with more complex shock/stimulus sequences to further enhance electrotherapy efficacy for monomorphic VTs (as tested here) and potentially also polymorphic VTs and VF.

### 4.5 Sensitivity analysis of the placement of transmural electrodes

The sensitivity tests of transmural electrodes are conducted in this study to investigate its feasibility and reliability for clinical usage in the future. We found that varying the location of ground electrodes reduces the efficacy of VT termination slightly to be 73% from 85%. This small decrease of efficacy is due to the difference in the placement of the ground electrode, producing different shock vectors which in turn affects the targeted area. As shown in Fig. 8 (a), for the transmural configuration, VT is terminated because the shock depolarizes a critical area of myocardium in the CI which prevents further VT propagation. In cases with different ground electrode placements, the critical area may not be fully depolarized by the altered shock vector, which could result in failure to terminate. The effect of electrode location can be controlled by carefully placing the electrodes with the aid of prior knowledge of VT substrate regions.

Instead of placing the electrodes at an optimal location, we also showed that a potentially more straightforward approach to ensure robustness of the transmural configuration may be to use larger electrodes i.e. the extended electrodes shown in Fig. 4, similar to a conventional coil used in ICDs. In addition, we also tested the case in which the ground electrode were moved out of the LV blood pool, while maintaining the shock vector targeted at the centre of the CI, which still ensured 100% efficacy on the small test cohort. Such a setup may be more achievable than having an electrode in the LV, utilising conventional RV electrodes and multipolar electrodes in LV veins of a CRT-D device.

### 4.6 Practical clinical utility of novel designs

In order to utilise the ability of directional stimulation of both novel electrodes, it is important to know approximate location of the VT substrate prior to implantation. Imaging techniques, such as contrast-enhanced MRI is the most commonly-used method to obtain such information. There are also clinical algorithms which can predict the approximate VT exit sites based on the electrocardiograms of VT [40]. Furthermore, we are currently working on real-time automated identification of VT locations using electrograms (EGMs) recorded from ICDs [41].

The bipolar electrode configuration has more complex electrodes which may require more radical overhaul of current clinical ICD electrode design and manufacture. However, this configuration may be modified as a single lead, where all the conducting surfaces are placed, similar to current RV coils used in present ICDs. Furthermore, similar bipolar designs with directional capability also exist in brain stimulation [42,43], demonstrating the potential feasibility to further our designs in the setting of cardiac electrotherapy. Practically placing a lead in the LV may be problematic due to implantation complexities and associated increasing risks of blood clot formation. In this cases, our bipolar configuration may also be placed in the RV which will still be effective to septal VTs, as shown in this study. For VTs in other locations of the LV, may still deliver directional shocking, albeit at an additional distance.

In contrast, the transmural electrode represents a more clinically translatable design in the short-term, as it may be incorporated into the design of current clinical devices (CRT-Ds), as shown in Fig. 4, utilising the RV coil and electrodes in the lead within the LV vein (coronary sinus). Moreover, novel designs are emerging which show the potential for transmural configurations to be further developed. For example [[44], [45], [46], [47]], describe novel electrotherapy electrode designs that are able to attach to the epicardial surface of the heart to provide highly localised electrical recording and stimulation. Such flexible electrodes may provide the distal electrode (outside the heart) in our transmural configuration, with the dis-similar intra-cavity electrode provided by a standard RV-coil electrode (as tested in our sensitivity analysis).

### 4.7 Limitations and future work

In this study we used a human ventricular ionic cell model, adjusted to replicate the expected porcine action potential duration. This was due to the absence of a porcine-specific cell model at the time the study was conducted. Unfortunately, it was not possible to perform the study with a cohort of human ventricular anatomical models, due to difficulties in obtaining high-resolution MRI of infarct patients of sufficient quality for detailed model construction. As mentioned, the ability of our models to faithfully reconstruct the detailed structural anatomy of the scar and BZ was essential for accurate representation of infarct-driven VT dynamics and would not have been possible with clinical data at the present time. Secondly, this study has a relatively small cohort of three porcine models (as monomorphic VT was non-inducible in the other four porcine models that are also developed in the same pipeline as shown in Fig. 1(a)). However, we were able to increase the variety of induced VT episodes in our 3 models by tuning EP properties, enabling both fast and slow VTs with a range of VTCLs observed clinically to be simulated (as in Ref. [22]). Thirdly, the scar regions in all three pig models was located near the septum, due to the specific infarct-induction procedure applied experimentally. The ATP delivery sites in this study were chosen as those commonly used in ICDs, delivered through the RV lead placed close to the septum near the apex. In reality, the scar can be located in a range of locations throughout both the LV, RV and septum. However, we believe that, as our novel configurations are ultimately designed to target the specific substrate sustaining the VT, assessing their efficacy in the models used here represents a robust approach.

The next step of this study will be to conduct rigorous experimental validation, which would pave the way to further optimisation of the proposed designs for manufacturing and production. The simulation work conducted here circumvents many inherent experimental limitations in performing such investigations (for example, difficulties in inducing stable, monomorphic VT, issues with fabricating bespoke electrode designs) and thus provides much utility to focus and refine the experimental parameters to be investigated and the scope of the experiments undertaken.

## 5 Conclusion

Two novel electrode configurations are proposed here that have the ability to deliver directional stimulation to the centre of a reentrant circuit, enhancing electrotherapy efficacy for VT termination, above conventional VT, tested on an in silico cohort of porcine infarcted LV models. The bipolar electrode configuration is able to confine the majority of the electric field within the heart, requiring similar shock energies to conventional ICD biphasic shocks, though importantly whilst reducing deposition of energy outside of the heart, potentially reducing unwanted ICD pain and side-effects. Our novel proposed transmural electrode configuration places electrodes physically closer to the targeted tissue, therefore able to achieve a very high efficacy with delivered energy two orders of magnitude lower than conventional ICD defibrillation. This work opens-up opportunities for further direct manufacture and experimental testing, as well as further in silico optimisation and testing, of these novel designs.

## Conflict of interest statement

The authors of the paper titled as ‘An in-silico assessment of efficacy of two novel intra-cardiac electrode configurations versus traditional anti-tachycardia pacing therapy for terminating sustained ventricular tachycardia’ declare that we have no known competing financial interests or personal relationships that could have appeared to influence the work reported in this paper.

## Declaration of competing interest

The authors declare that there is no conflict of interest.
