# Supplementary material for: An in-silico assessment of efficacy of two novel intra-cardiac electrode configurations versus traditional anti-tachycardia pacing therapy for terminating sustained ventricular tachycardia
Source: Comput Biol Med. 2021 Dec;139:104987. doi: 10.1016/j.compbiomed.2021.104987 (PMC8669079; doi:10.1016/j.compbiomed.2021.104987)
Supplement: Multimedia component 1 [file mmc1.docx]

An in-silico assessment of efficacy of two novel intra-cardiac electrode configurations versus traditional anti-tachycardia pacing (ATP) therapy for terminating sustained ventricular tachycardia

**A. Appendix**

Here shows the full results of individual strengths required by both bipolar and transmural configurations to terminate all six VTs as in Figure S1. As is shown, the strength required for terminating VTs varies significantly in different VTs. In Figure S1(a), it shows that for bipolar configuration, at the delivery time of 88% VTCL, the strength to achieve partially high efficacy $S_{\mathrm{pEff}}$ is 860 V, while the strength to achieve full efficacy $S_{\mathrm{fEff}}$ is 1700 V. The strengths $S_{\mathrm{pEff}}$ and $S_{\mathrm{fEff}}$ for transmural configuration are 80 V and 120 V as shown in Figure S1(b).


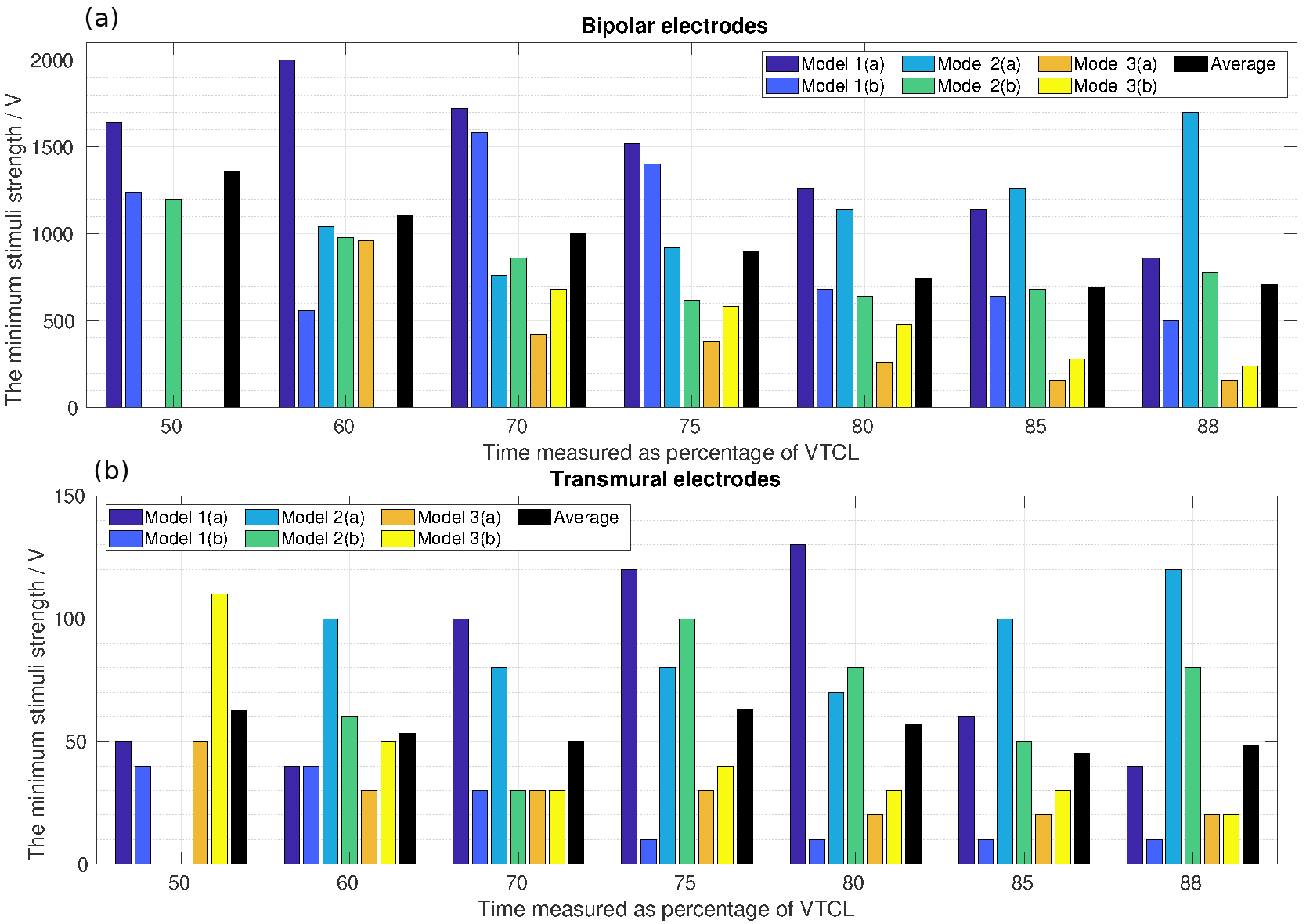


Figure S1: Minimum stimulus strength needed for terminating VT in (panel a) bipolar electrodes and (panel b) transmural electrodes were plotted against the pacing time defined as the percentage of the VTCL since the last activation detected at the CI. Six VTs were tested and their average minimum stimulus strength at each pacing times were shown in black. Noted that at 50% and 60% of VTCL, some VTs were not terminated even the stimulus strength increasing until the computational simulation crashed. Therefore, the average value is calculated by eliminating those unsuccessful cases.
